# A note on statistical repeatability and study design for high‐throughput assays

**DOI:** 10.1002/sim.7175

**Published:** 2016-11-24

**Authors:** George Nicholson, Chris Holmes

**Affiliations:** ^1^ Department of StatisticsUniversity of Oxford24‐29 St GilesOxfordOX1 3LBU.K.

**Keywords:** high‐throughput assay, technical replicate, scatterplot, study design, repeatability

## Abstract

Characterizing the technical precision of measurements is a necessary stage in the planning of experiments and in the formal sample size calculation for optimal design. Instruments that measure multiple analytes simultaneously, such as in high‐throughput assays arising in biomedical research, pose particular challenges from a statistical perspective. The current most popular method for assessing precision of high‐throughput assays is by scatterplotting data from technical replicates. Here, we question the statistical rationale of this approach from both an empirical and theoretical perspective, illustrating our discussion using four example data sets from different genomic platforms. We demonstrate that such scatterplots convey little statistical information of relevance and are potentially highly misleading. We present an alternative framework for assessing the precision of high‐throughput assays and planning biomedical experiments. Our methods are based on repeatability—a long‐established statistical quantity also known as the intraclass correlation coefficient. We provide guidance and software for estimation and visualization of repeatability of high‐throughput assays, and for its incorporation into study design. © 2016 The Authors. Statistics in Medicine Published by John Wiley & Sons Ltd.

## 1 Introduction

In the post‐genome era, assays such as sequencing technologies and microarrays have underpinned major advances in biomedical genetics and form key components of recent large‐scale projects in medical science, such as the Precision Medicine Initiative 1 and the 100 000 Genomes Project 2. In recent years, the number of analytes measurable in a single experiment has increased dramatically, broadening the scope of scientific studies while raising new questions on the reproducibility of their conclusions 3, 4, 5, 6. While there has been extensive work on post‐experimental statistical procedures for controlling false discovery rates 6, 7, 8, little guidance exists on how to assess the precision of multivariate assays and incorporate this into experimental study design and the planning of experiments. Here, we critically review the current standard practice of quantifying assay performance, which is to calculate the sample correlation of measurements across a pair of multivariate technical replicates 9, 10, 11, 12, 13, 14, 15. We highlight important flaws in this approach and present an alternative framework based on statistical repeatability (also known as the intraclass correlation coefficient), for communicating assay precision and for integrating it into the planning of high‐throughput experiments 16.

In their influential work on measuring the agreement between two medical instruments 17, 18, 19, Bland and Altman (BA) challenged the convention of scatterplotting the *univariate* data of one instrument against the other, that is, one point per patient, and of interpreting high correlation as indicating agreement between instruments. Our work can be thought of as extending these existing ideas of correlation and repeatability to a high‐throughput multivariate‐measurement setting, where a single instrument is used to measure multiple analytes on a set of individuals. Moreover, we pay particular attention to the issue of optimal experimental design for high‐throughput assays.

## 2 Correlation between repeated measures as an indication of assay precision.

**Figure 1:**
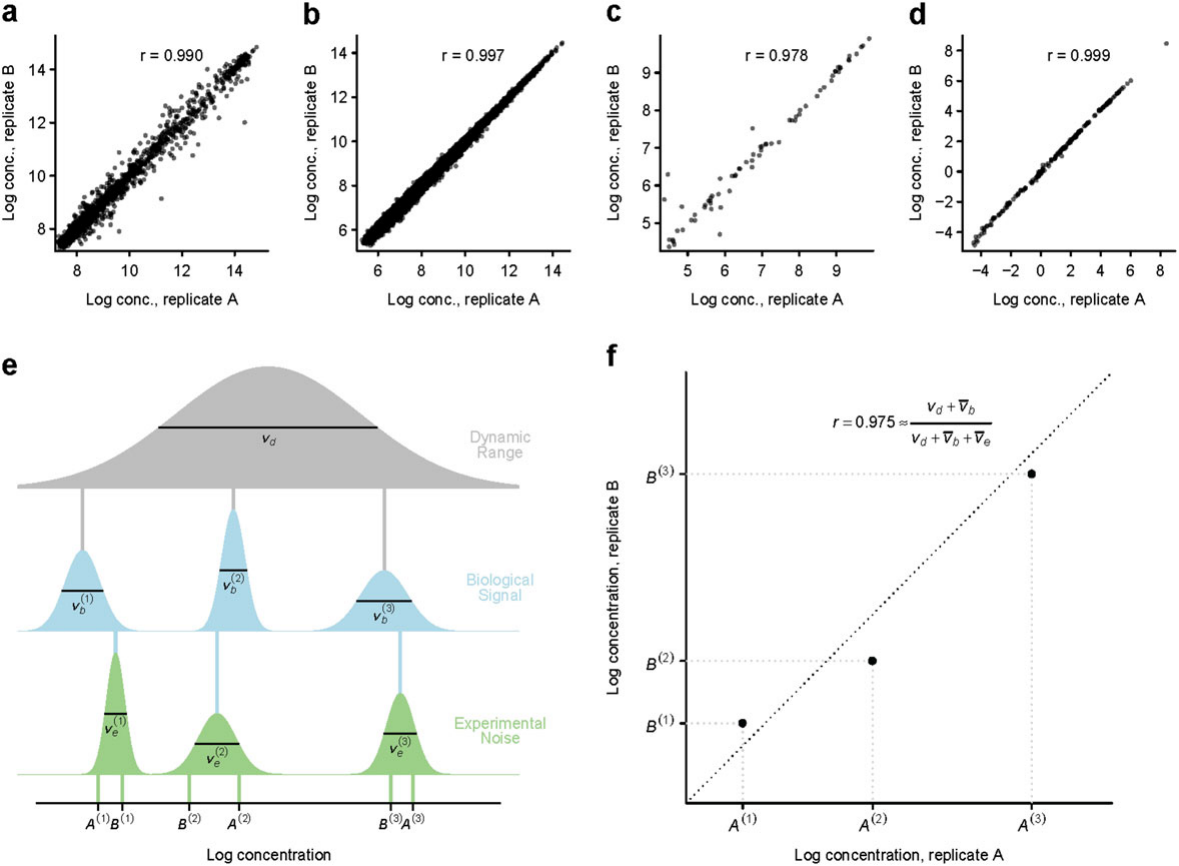
Scatter plots of technical replicates—examples and underlying statistical model. **(a–d)**(a–d) Scatter plots of measured log concentrations from two technical replicates on each of four high‐throughput assays (Table 1). Each point displays the two replicate measurements of a particular analyte's concentration. Pearson's sample correlation coefficient, r, is shown. One pair of replicates was chosen at random from each data set (distribution of r across all pairs is shown in Figure S1). **(e)**(e) Sources of variation underlying a pair of technical replicates. The grey bell‐shaped distribution represents variation in concentration across analytes, spanning the entire dynamic range of the assay with dynamic‐range variance v_d_. Three analytes, labelled 1–3, are drawn from this distribution, and their population‐mean concentrations are represented by vertical grey lines. The blue distributions represent variation in concentration across a population of individuals around the population's mean, represented by analyte‐specific biological signal variances $v_{b}^{\left(1\right)},v_{b}^{\left(2\right)},v_{b}^{\left(3\right)}$, with the average of these biological variances across analytes denoted by ${\bar{v}}_{b}=\frac{1}{3}\left(v_{b}^{\left(1\right)}+v_{b}^{\left(2\right)}+v_{b}^{\left(3\right)}\right)$. A particular individual's concentrations at the three analytes, represented by vertical blue lines, are drawn from these distributions. The green distributions represent measurement error around the individual's true concentrations, with analyte‐specific experimental noise variances $v_{e}^{\left(1\right)},v_{e}^{\left(2\right)},v_{e}^{\left(3\right)}$, with the average of these experimental variances across analytes denoted by ${\bar{v}}_{e}=\frac{1}{3}\left(v_{e}^{\left(1\right)}+v_{e}^{\left(2\right)}+v_{e}^{\left(3\right)}\right)$. A pair of technical replicates, A and B, with data labelled (A ^(1)^,A ^(2)^,A ^(3)^) and (B ^(1)^,B ^(2)^,B ^(3)^), are drawn from the green distributions and shown at the base of the plot. **(f)**(f) Scatter plot comparing the technical replicates' data from **e**e.

A common means of reporting the precision of a high‐throughput (also known as multiplex or high‐content) assay in the literature is to compare a pair of *technical replicates*, such as those obtained by splitting a biological sample into two aliquots, and analysing each aliquot separately on the assay. The two technical replicates, each comprising measurements from multiple analytes, are plotted against each other, one point per analyte, and the corresponding sample correlation coefficient, *r*, is reported as a measure of experimental precision; see for example 9, 10, 11, 12, 13, 14, 15. As illustration, Figure 1a–d displays this method applied to a pair of replicates from each of four representative high‐throughput assays 20, 21, 22, 23.

The intuition behind these plots is simple: a ‘high‐precision assay’ has little variation in repeated measurements on the same sample, a property that is represented graphically by points lying close to the diagonal *x* = *y* line, and statistically by large inter‐replicate sample correlation of *r*≈1. This intuition is correct, in that extremely precise assays necessarily result in *r*≈1. However, the commonly employed argument that an assay exhibiting *r*≈1 implies an extremely precise measurement is, somewhat unintuitively, false. The reason is that the assay's dynamic range across analytes is confounded with *r* when considered as a measurement of experimental precision.

**Table 1:** Assay details, sample size and estimated components of variance.

			Number of samples	**Estimated variance** (95% CI)				Estimated *R* ^(*k*)^
Assay	Target	Number of analytes	(**Number replicated**)	*v*_*d*_	${\bar{v}}_{b}$	${\bar{v}}_{e}$	*r*	**Median** (IQR) across *k*
a	microRNAs	1624	69 (**69**)	**3.57** (3.54–3.59)	**0.05** (0.04–0.05)	**0.07** (0.06–0.07)	.990	**0.31** (0.10–0.53)
b	mRNAs	17 788	76 (**15**)	**1.95** (1.95–1.96)	**0.03** (0.02–0.03)	**0.01** (0.01–0.01)	.997	**0.59** (0.24–0.80)
c	Proteins	69	215 (**45**)	**2.47** (2.45–2.50)	**0.02** (0.02–0.03)	**0.06** (0.05–0.07)	.978	**0.31** (0.20–0.50)
d	Metabolites	163	287 (**67**)	**8.17** (8.13–8.21)	**0.04** (0.04–0.05)	**0.01** (0.01–0.01)	.999	**0.94** (0.82–0.96)

## 3 Statistical analysis using a variance components model

To understand better the phenomenon described, it is helpful to consider a multilevel statistical model for the data. We utilize a model to decompose the variation underlying concentrations of the *p* analytes measured in technical replicate on each of several biological samples as

(1) $$y_{ij}^{\left(k\right)}=\mu +a^{\left(k\right)}+b_{i}^{\left(k\right)}+e_{ij}^{\left(k\right)},$$

where $y_{ij}^{\left(k\right)}$ is the measured concentration of the *k*th analyte in the *j*th replicate of the *i*th biological sample, and *μ* is the global mean concentration. The *a* ^(*k*)^, $b_{i}^{\left(k\right)}$ and $e_{ij}^{\left(k\right)}$ are independent zero‐mean random variables contributing components of variance, with *v*_*d*_ ≡ *V*(*a* ^(*k*)^) as the *dynamic range* variance in concentration across analytes; $v_{b}^{\left(k\right)}\equiv V\left(b_{i}^{\left(k\right)}\right)$ as the *biological signal* variance across individuals at the *k*th analyte; and $v_{e}^{\left(k\right)}\equiv V\left(e_{ij}^{\left(k\right)}\right)$ as the *experimental noise* variance at the *k*th analyte.

Using the variance‐component model, we are then able to relate the empirical sample correlation *r* to physical sources of variation. In particular, we are led to the following result,

Proposition 1 (2) $$r\overset{Pr}{\to }\frac{v_{d}+{\bar{v}}_{b}}{v_{d}+{\bar{v}}_{b}+{\bar{v}}_{e}}$$

where $\overset{Pr}{\to }$ denotes convergence in probability as the number of analytes measured *p*→*∞*, and where ${\bar{v}}_{b}=\frac{1}{p}\sum_{k}v_{b}^{\left(k\right)}$, ${\bar{v}}_{e}=\frac{1}{p}\sum_{k}v_{e}^{\left(k\right)}$. The proof is contained in Supporting Information Appendix A.

To examine the finite‐sample behaviour of (2), we performed a re‐sampling study of the four data sets, concluding that *r* converges to within 1*%* of its final value by *p*≈100 (data not shown). Formula (2) reveals that *r* is close to 1 whenever the average noise term ${\bar{v}}_{e}$ is small relative to the sum of the dynamic range and average signal terms $v_{d}+{\bar{v}}_{b}$. In particular, to attain high correlation, it is not necessary for the assay's noise to be small relative to its signal, provided its noise is small relative to its dynamic range. This effect is illustrated in Figure 1e,f, where the noise variances $v_{e}^{\left(k\right)}$ are small relative to the dynamic range *v*_*d*_, leading to high‐sample correlation of *r* = 0.975, despite the noise $v_{e}^{\left(k\right)}$ and signal $v_{b}^{\left(k\right)}$ being of comparable size.

Returning to the four data sets 20, 21, 22, 23 introduced in Figure 1a–d, we estimated their corresponding variance components directly on each full set of data (Table 1). We found each assay's average noise variance ${\bar{v}}_{e}$ to be of a similar magnitude to its signal ${\bar{v}}_{b}$, but two to three orders of magnitude smaller than its dynamic range *v*_*d*_. This demonstrates empirically that these assays exhibit considerable levels of noise (relative to biological signal ${\bar{v}}_{b}$) while achieving high inter‐replicate correlation, as in Figure 1a–d, because their dynamic range is wide. Our advice is to avoid scatterplotting or calculating *r* between pairs of technical replicates, as such tools provide little statistical information on quantities of interest when correctly interpreted, and can be severely misleading when misinterpreted.

## 4 Repeatability of high‐throughput assays and its use in study design

Instead, we suggest an approach for characterizing the precision of high‐throughput assays, and for integrating that information into the planning of well powered experiments. Our recommendation is based on the repeatability, a long‐established statistical quantity, also known as the intraclass correlation coefficient, reviewed in 24. The repeatability at analyte *k* is defined as

(3) $$R^{\left(k\right)}:=\frac{v_{b}^{\left(k\right)}}{v_{b}^{\left(k\right)}+v_{e}^{\left(k\right)}}$$

where the analyte's biological signal variance $v_{b}^{\left(k\right)}$ and experimental noise variance $v_{e}^{\left(k\right)}$ are defined in Figure 1e and its legend, and at the beginning of Section 3. The repeatability is a quantity in the interval [0,1] that records the proportion of total observed variance at an analyte that is attributable to biological sources. At the upper end of the scale, *R* ^(*k*)^ = 1 indicates that analyte *k* is measured perfectly with $v_{e}^{\left(k\right)}=0$ while, at the lower end, *R* ^(*k*)^≈0 signifies data that are dominated by experimental variability with $v_{e}^{\left(k\right)}\gg v_{b}^{\left(k\right)}$.

**Figure 2:**
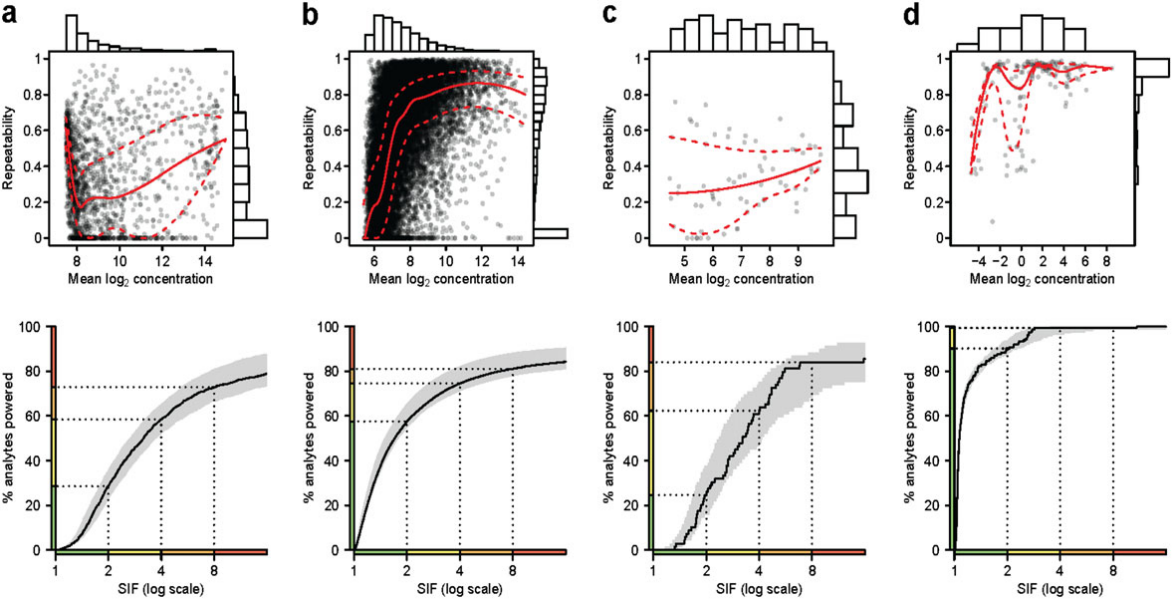
Proposed graphical representations of assay precision. **(a–d)**(a–d) Repeatability versus concentration scatter plot (top) and plot of cumulative % of analytes powered (bottom), for four high‐throughput assays (Table 1). **Top panels**Top panels: Scatter plot of repeatability R against mean measured log_2_ concentration (one point per analyte). To visualize dependence of repeatability on concentration, median (red solid line) and quartiles (red dashed lines) of repeatability are plotted as a smooth function of concentration. The histogram at right shows the distribution of R across analytes, and the histogram at top shows the distribution of mean measured log_2_ concentration across analytes. **Bottom panels**Bottom panels: the black line shows the effect of increasing the sample size inflation factor, SIF, on the % of analytes powered to detect an effect. Grey‐shaded regions are 95% bootstrap confidence intervals for the black line (details in the Supporting Information Appendix C). Intervals on the horizontal axis are coloured according to SIF and are mapped to the vertical axis for reference.

Analyte repeatabilities can be estimated directly under a standard pilot study that incorporates technical replicates (pilot design recommendations are provided in the Appendix). Potential estimation methods include analysis of variance (ANOVA), maximum likelihood and restricted maximum likelihood 24, 25. Here, we choose ANOVA‐based estimators because they are available in closed form, leading to computationally efficient implementation of the parametric bootstrap 26 used to calculate confidence intervals (Figure 2 bottom panels; Supporting Information Appendix B). ANOVA estimators for variance parameters can take negative values. In particular, it is possible that ${\hat{v}}_{b}^{\left(k\right)}<0$, while it is known that $v_{b}^{\left(k\right)}⩾0$. We set negative variance estimates to zero, leading to upwards bias but a net decrease in mean‐squared error (25, their Section 4.4).

Bland and Altman (BA) proposed the calculation of the ‘repeatability coefficient’ for a single instrument 18. BA's repeatability coefficient ( $R_{BA}\equiv 1.96\sqrt{2}{\hat{v}}_{e}$ in our notation) provides a 95% one‐sided upper bound for the absolute difference between a pair of replicate readings on the instrument. *R*_*BA*_, being on the same scale as the instrument itself, has the advantage of allowing simple clinical assessment of true biological changes 18, 27, but does not incorporate information on the biological variation across subjects, *v*_*b*_. The repeatability as defined at ((3)) (i.e. the intraclass correlation coefficient, ICC) is a dimensionless quantity targeting the proportion of variation in an instrument's measurements that arises from non‐experimental sources. We advocate the ICC for the purposes of assessing the repeatability of a high‐throughput assay, for it is advantageous to have a measure of repeatability that is both scale‐free (allowing direct pooling of information across analytes) and that incorporates *v*_*b*_, which, together with *v*_*e*_, is necessary for considerations of experimental design.

It is often the case that measurement precision shows a relationship with analyte concentration; for example, it can be relatively difficult to measure the abundance of low‐concentration analytes. We recommend a scatter plot of estimated repeatability at each analyte against that analyte's average measured concentration to highlight any association (Figure 2, top panels). The distribution of repeatability estimates is visualized effectively as a histogram, as on the right edge of the top plots in Figure 2. Distributional summaries, such as median and inter‐quartile range (Table 1 final columns), can be usefully reported when space is limited, although these particular statistics do not summarize the data distribution effectively in all cases; for example, they are not good summaries of assay b's bimodal repeatability distribution (Figure 2b top panel).

### 4.1 Illustrations and sample‐size calculation

To illustrate the application of repeatability to study design, we first consider a sample size calculation for an experiment performed using a perfect instrument, and then show how that sample size should be increased on the basis of repeatability to ensure power is attained in the presence of measurement error.

Consider an experiment aimed at identifying differences in analyte concentration between treatment and control groups. Let *μ*_*T*_ denote the true underlying mean for the treatment group, and *μ*_*C*_ the true mean for the control group. To calculate sample size requirements, the key quantity to specify is the standardized effect size, $\Delta \;\equiv \;\frac{\left|\mu_{T}-\mu_{C}\right|}{\sqrt{v_{b}}}$, that is, the absolute difference between groups in units of the biological standard deviation $\sqrt{v_{b}}$. For a simple example, consider a user‐specified targeted effect size of Δ = 1, with power required to be 80*%* at a false‐positive rate of 0.05. The resulting calculation indicates that *n*_0_ = 34 participants are required, 17 in each group, to be powered to detect the specified effect on a perfect instrument (see 16 for a useful introduction to power and sample size).

In practice, instead of having a perfect instrument with repeatability 1, each analyte *k* on an assay is actually measured with its own particular non‐zero measurement error $v_{e}^{\left(k\right)}>0$ and hence repeatability *R* ^(*k*)^ < 1. The experimenter might choose a single sample size *n* that applies to all analytes on the assay. It is intuitively desirable to choose *n* larger than the sample size for a perfect instrument, *n*_0_, to compensate for measurement error being present. One way of characterizing the increase in chosen sample size relative to that of a perfect instrument is the ratio *n*/*n*_0_ which we define as the *sample size inflation factor* (SIF),

$$\text{SIF}:=\frac{n}{n_{0}}\equiv \frac{\text{sample size required for assay with measurement error}}{\text{sample size required by perfect instrument}}\;.$$

The distribution of repeatabilities across an assay provides a framework for informed choice of SIF. In particular, we are able to state the following result.

Proposition 2 The experiment is well powered to detect changes in the expected value of analyte *k* if

$$\text{SIF}>\frac{1}{R^{\left(k\right)}}\;.$$

The proof is given in the Supporting Information Appendix C.

*Proposition* 2 provides a basis for taking the sample size required by a perfect instrument (*n*_0_) and inflating it to a sample size suitable for an assay with measurement error (*n*), so that the experiment is powered at a specified proportion of analytes. Our proposed protocol for the design of a high‐throughput experiment aimed at detecting mean differences in analyte concentration between two groups is thus as follows.

1. Estimate *R* ^(*k*)^ at analytes *k* = 1,…,*p*, based on data from a pilot experiment with samples assayed in technical replicate.
2. Select SIF large enough so that a user‐specified proportion of analytes on the assay satisfy SIF > 1/*R* ^(*k*)^ and are hence powered. In practice, this step is best performed with reference to plots and tables based on assay‐wide repeatability estimates such as Figure 2 bottom panels, and Table 2.
3. Specify the experiment's targeted standardized effect size Δ, nominal significance level *α*, and power, and use them to calculate the sample size, *n*_0_, required by a perfect instrument. ‡Step 3 can be performed using any standard power software, such as G^∗^Power 28 or the function power.t.test() in R 29. Note that if statistical tests are to be performed at each of a large number of analytes then the specified significance level *α* should be correspondingly more stringent. For example, Bonferroni adjustment could be used to control the family‐wise error rate across all analytes tested.
4. Calculate the adjusted sample size as *n* = SIF × *n*_0_

**Table 2:** Percentage of analytes powered for different SIF values.

	**Percentage of analytes powered** (95% CI)			
SIF	Assay a	Assay b	Assay c	Assay d
1.1	**1** (0–1)	**10** (9–15)	**0** (0–0)	**60** (56–64)
1.5	**11** (8–16)	**42** (39–49)	**3** (3–13)	**83** (82–86)
2	**29** (23–35)	**58** (55–65)	**25** (14–39)	**90** (88–94)
3	**48** (42–54)	**69** (67–77)	**45** (38–62)	**99** (93–99)
4	**58** (53–64)	**75** (72–82)	**62** (49–74)	**99** (96–100)
5	**65** (59–70)	**77** (75–85)	**74** (57–81)	**99** (97–100)

Software in R for estimating and visualizing assay‐wide repeatabilities (as per Figure 2 and Table 2) from data sets with technical replicates is freely available on request.

Hence, as SIF is increased, the % of analytes that are powered increases accordingly. By quantifying and inspecting this relationship (Figure 2, bottom panels; Table 2), the user can control the *%* of analytes at which an experiment is powered by varying SIF. For assays a, b, c, and d to be powered at approximately 60% of analytes, suitable SIFs would be 4, 2, 4, and 1.1 respectively (Table 2), translating into sample sizes of 136, 68, 136 and 38 when applied to the sample‐size calculation above with *n*_0_ = 34. When designing a study, in addition to reporting *n*_0_ and its calculation based on Δ, *α* and power, we suggest reporting the selected SIF and adjusted sample size *n*, along with the corresponding point estimate and confidence interval for the % of analytes powered (Table 2).

It is natural to consider SIF as a form of variance inflation factor. VIFs measure collinearity amongst explanatory variables in multiple linear regression, reflecting the multiplicative increase in $V({\hat{\beta }}_{j})$ due to non‐zero correlations between *x*_*j*_ and the other covariates 30. VIFs can also be used to inflate sample sizes calculated under basic two‐group designs so that they apply to more complex design settings 31. At analyte *k*, the VIF

(4) $$\frac{1}{R^{\left(k\right)}}\equiv \frac{v_{b}^{\left(k\right)}+v_{e}^{\left(k\right)}}{v_{b}^{\left(k\right)}}$$

is the multiplicative increase in $V({\hat{\beta }}_{j})$(for all *j*) for the model $y∼N\left(X\beta ,(v_{b}^{\left(k\right)}+v_{e}^{\left(k\right)})I\right)$ relative to the model $y∼N(X\beta ,v_{b}^{\left(k\right)}I)$, with Proposition 2 demonstrating that this VIF can be used to inflate sample size appropriately in the balanced two‐group setting.

## 5 Conclusion

In conclusion, when designing high‐throughput experiments, it is important to quantify those aspects of assay precision that relate directly to the study objectives. We have shown empirical and theoretical evidence that the standard approach of communicating assay precision—via correlation and scatterplotting of data from technical replicates—provides little statistical information at best and is often misleading. We have presented alternative statistical methods based on the notion of analyte repeatability, quantifying the information in an assay relative to a perfect instrument and providing a framework for adjusting sample size accordingly.

## Data Accessibility

## Supporting information



Supporting info item

## Appendix A

This appendix contains guidance on the design of pilot studies aimed at estimating repeatability and also more practical guidance on choosing SIF for main studies.

### A1 Sample size for a pilot study

For choice of sample size under model (1), our suggestion is to focus on achieving effective estimation of the distribution of repeatabilities across all analytes, as opposed to the repeatability for any particular analyte. This is because it is typically unknown in advance which of the assayed analytes will be of eventual interest, and so it is natural to plan experiments based on the whole set. Also, a relatively large number—of the order of hundreds—of replicated samples is required to obtain precise repeatability estimates for individual analytes (32, their Figure 3).

To assess what sample size is sufficient for estimating the distribution of repeatabilities, we repeatedly randomly sub‐sampled and re‐analysed each of the four example data sets. Each sub‐data set comprised a number of samples assayed in technical duplicate, denoted by *D*∈{3,6,9,12}, and a number of samples assayed only once, denoted by *S*∈{0,6,12,18,24}. The resulting plots of cumulative *%* of analytes powered are shown in the Figures S2–5. The feature of interest in these plots is the reduction in width of confidence interval with increasing sample size. It appears possible to reduce technical replication in the pilot study to quite a low level, for example just three replicated samples, provided that an adequate number of assays is conducted in total. Our suggestion is to perform at least 20 assays in the pilot study, with at least three samples assayed in technical duplicate (in the above notation, D⩾3 with 2D+S⩾20).

### A2 Choice of SIF for a main study

In choosing suitable SIF, it is important to take into account the confidence intervals (CIs) for % of analytes powered, as shown in Figure 2 (bottom panels) and Table 2. It is especially important in cases where the CIs are wide, for example when only a small number of pairs of replicates is assayed in the pilot study (Figures S2–5). If it is essential that a minimum % of analytes is powered, then SIF can be selected to be large enough that the lower bound of the CI exceeds the required %.

For a study in which a particular subset of analytes is of primary interest (e.g. measurements related to genes in a particular pathway), the SIF can be chosen to ensure that some proportion *p*_1_ of the subset is powered, while a different proportion *p*_2_ of all analytes on the array is powered. Creating such a design would involve applying our methods twice, once to the subset and once to the global set of analytes. SIF would be chosen to be the maximum of SIF_1_ and SIF_2_, where SIF_1_ powers *p*_1_ of the subset, and SIF_2_ powers *p*_2_ of all analytes.
