# Supplementary material for: A note on statistical repeatability and study design for high‐throughput assays
Source: Stat Med. 2016 Nov 24;36(5):790–8. doi: 10.1002/sim.7175 (PMC5299465; doi:10.1002/sim.7175)
Supplement: Supplementary file 1 — Supporting info item [file SIM-36-790-s001.zip › supplementary_information_nicholson_holmes.pdf]

Supporting Information

3 **Appendices** and 5 **Supplementary Figures**

Supporting Information Appendix A

Here we detail the proof of **Proposition 1**, which is essentially an application of the multivariate delta method.

Lemma 1. *If* $\left\{ \begin{pmatrix} z_1^{(k)} \\ z_2^{(k)} \end{pmatrix} : k = 1, \ldots, p \right\}$ *is a sequence of i.i.d. random variables with covariance matrix* $\begin{pmatrix} v & \tau \\ \tau & v \end{pmatrix}$, $v > 0$, $E\left[\left(z_j^{(k)}\right)^4\right] < \infty$, *and*

$$r \;:=\; \frac{\sum_{k=1}^{p}\left(z_1^{(k)} - \bar{z}_1^{(\cdot)}\right)\left(z_2^{(k)} - \bar{z}_2^{(\cdot)}\right)}{\sqrt{\sum_{k=1}^{p}\left(z_1^{(k)} - \bar{z}_1^{(\cdot)}\right)^2 \sum_{k=1}^{p}\left(z_2^{(k)} - \bar{z}_2^{(\cdot)}\right)^2}}, \tag{5}$$

then

$$r \overset{Pr}{\to} \frac{\tau}{v} \text{ as } p \to \infty.$$

Proof. The i.i.d. property and finite-moment assumptions allow application of the weak law of large numbers (WLLN) to show that (as $p \to \infty$)

$$\begin{aligned}
\frac{1}{p}\sum_{k=1}^{p}\left(z_1^{(k)} - \bar{z}_1^{(\cdot)}\right)\left(z_2^{(k)} - \bar{z}_2^{(\cdot)}\right) &= \frac{1}{p}\sum_{k=1}^{p}\left(z_1^{(k)} z_2^{(k)}\right) - \left(\frac{1}{p}\sum_{k=1}^{p} z_1^{(k)}\right)\left(\frac{1}{p}\sum_{k=1}^{p} z_2^{(k)}\right) \\
\text{[by WLLN and Slutsky's theorem]} &\overset{Pr}{\to} E\left[z_1^{(1)} z_1^{(2)}\right] - E\left[z_1^{(1)}\right] E\left[z_1^{(2)}\right] \\
&= \tau
\end{aligned} \tag{6}$$

and that, for $j \in \{1, 2\}$,

$$\begin{aligned}
\frac{1}{p}\sum_{k=1}^{p}\left(z_j^{(k)} - \bar{z}_j^{(\cdot)}\right)^2 &= \frac{1}{p}\sum_{k=1}^{p}\left(z_j^{(k)}\right)^2 - \left(\frac{1}{p}\sum_{k=1}^{p} z_j^{(k)}\right)^2 \\
\text{[by WLLN]} &\overset{Pr}{\to} E\left[\left(z_j^{(1)}\right)^2\right] - E\left[z_j^{(1)}\right]^2 \\
&= v.
\end{aligned} \tag{7}$$

Combining (5), (6), (7), and $v > 0$ with Slutsky's theorem gives

$$r \overset{Pr}{\to} \frac{\tau}{v} \text{ as } p \to \infty.$$

$\square$

Proposition 1. *If*

$$y_{ij}^{(k)} = \mu + a^{(k)} + b_i^{(k)} + e_{ij}^{(k)},$$

where

- $\{a^{(k)} : k = 1, \ldots, p\}$ *are i.i.d. random variables with* $E\left[a^{(k)}\right] = 0$, $V\left(a^{(k)}\right) = v_d$ *(with* v_d *non-random), and* $E\left[\left(a^{(k)}\right)^4\right] < \infty$,
- $\{b_i^{(k)} : i = 1, \ldots, n\}$ *are i.i.d. random variables with* $E\left[b_i^{(k)}\right] = 0$, $V\left(b_i^{(k)} \mid v_b^{(k)}\right) = v_b^{(k)}$, *and* $E\left[\left(b_i^{(k)}\right)^4\right] < \infty$,
- $\{v_b^{(k)} : k = 1, \ldots, p\}$ *are i.i.d. random variables with* $E\left[v_b^{(k)}\right] = \bar{v}_b$,
- $\left\{e_{ij}^{(k)} : i = 1, \ldots, n, \ \ j = 1, 2\right\}$ *are i.i.d. random variables with* $E\left[e_{ij}^{(k)}\right] = 0$, $V\left(e_{ij}^{(k)} \mid v_e^{(k)}\right) = v_e^{(k)}$, *and* $E\left[\left(e_{ij}^{(k)}\right)^4\right] < \infty$,
- $\{v_e^{(k)} : k = 1, \ldots, p\}$ *are i.i.d. random variables with* $E\left[v_e^{(k)}\right] = \bar{v}_e$, *and*
- $v_d + \bar{v}_b + \bar{v}_e > 0$,

and r *is defined as:*

$$r := \frac{\sum_{k=1}^{p}\left(y_{11}^{(k)} - \bar{y}_{11}^{(\cdot)}\right)\left(y_{12}^{(k)} - \bar{y}_{12}^{(\cdot)}\right)}{\sqrt{\sum_{k=1}^{p}\left(y_{11}^{(k)} - \bar{y}_{11}^{(\cdot)}\right)^2 \sum_{k=1}^{p}\left(y_{12}^{(k)} - \bar{y}_{12}^{(\cdot)}\right)^2}}$$

then

$$r \overset{Pr}{\to} \frac{v_d + \bar{v}_b}{v_d + \bar{v}_b + \bar{v}_e} \quad \textit{as} \quad p \to \infty.$$

Proof. Note that

$$r = \frac{\sum_{k=1}^{p}\left(z_{11}^{(k)} - \bar{z}_{11}^{(\cdot)}\right)\left(z_{12}^{(k)} - \bar{z}_{12}^{(\cdot)}\right)}{\sqrt{\sum_{k=1}^{p}\left(z_{11}^{(k)} - \bar{z}_{11}^{(\cdot)}\right)^2 \sum_{k=1}^{p}\left(z_{12}^{(k)} - \bar{z}_{12}^{(\cdot)}\right)^2}}$$

where

$$z_{ij}^{(k)} := y_{ij}^{(k)} - \mu$$

so that $\begin{pmatrix} z_{11}^{(k)} \\ z_{12}^{(k)} \end{pmatrix} = \begin{pmatrix} a^{(k)} + b_1^{(k)} + e_{11}^{(k)} \\ a^{(k)} + b_1^{(k)} + e_{12}^{(k)} \end{pmatrix}$, and $E\left[\begin{pmatrix} z_{11}^{(k)} \\ z_{12}^{(k)} \end{pmatrix}\right] = \begin{pmatrix} 0 \\ 0 \end{pmatrix}$.

Also,

$$\begin{aligned} V\left(z_{11}^{(k)}\right) &= V\left(E\left[z_{11}^{(k)} \mid v_b^{(k)}, v_e^{(k)}\right]\right) + E\left[V\left(z_{11}^{(k)} \mid v_b^{(k)}, v_e^{(k)}\right)\right] \\ &= E\left[v_d + v_b^{(k)} + v_e^{(k)}\right] \\ &= v_d + \bar{v}_b + \bar{v}_e \end{aligned}$$

and

$$\begin{aligned} cov(z_{11}^{(k)}, z_{12}^{(k)}) &= E\left[z_{11}^{(k)} z_{12}^{(k)}\right] - E\left[z_{11}^{(k)}\right] E\left[z_{12}^{(k)}\right] \\ &= E\left[E\left(z_{11}^{(k)} z_{12}^{(k)} \mid v_d, v_b^{(k)}\right)\right] \\ &= E\left[v_d + v_b^{(k)}\right] \\ &= v_d + \bar{v}_b. \end{aligned}$$

Collecting these results gives that $\left\{\begin{pmatrix} z_{11}^{(k)} \\ z_{12}^{(k)} \end{pmatrix} : k = 1, \ldots, p\right\}$ are i.i.d. random variables each with covariance matrix

$$\begin{pmatrix} v_d + \bar{v}_b + \bar{v}_e & v_d + \bar{v}_b \\ v_d + \bar{v}_b & v_d + \bar{v}_b + \bar{v}_e \end{pmatrix},$$

and with $E\left[\left(z_{ij}^{(k)}\right)^4\right] < \infty$. Application of Lemma 1 gives:

$$r \stackrel{Pr}{\to} \frac{v_d + \bar{v}_b}{v_d + \bar{v}_b + \bar{v}_e} \text{ as } p \to \infty.$$

□

Supporting Information Appendix B

Here we detail the computation of bootstrap confidence intervals for the distribution function of repeatabilities across analytes. Data from high-throughput molecular assays usually exhibit strong correlation structure across analytes—a manifestation of biological relationships between the underlying molecules, as well as potential multivariate experimental artefacts. An important component of the parametric bootstrap [26] in this context is that the multivariate bootstrap distribution mimics the inter-analyte correlation structure observed in the true data.

The empirical CDF of the $R^{(k)}$, $k = 1, \ldots, p$, is

$$F(R) \equiv \frac{1}{p} \sum_{k=1}^{p} I\left(R \leq R^{(k)}\right) \ , \quad R \in [0, 1] \tag{8}$$

and is estimated by

$$\hat{F}(R) \equiv \frac{1}{p} \sum_{k=1}^{p} I\left(R \leq \hat{R}^{(k)}\right) \ , \quad R \in [0, 1] \ , \tag{9}$$

where $I(\cdot)$ is the indicator function. The precision of $\hat{F}(R)$ as an estimator of $F(R)$ is quantified by a bootstrap confidence interval [26].

Define, based on terms in (1),

$$\boldsymbol{y}_{ij} := \begin{pmatrix} y_{ij}^{(1)} \\ \vdots \\ y_{ij}^{(p)} \end{pmatrix} \ ,$$

and let the $n \times p$ matrix, $\boldsymbol{Y}_1$, comprise a single replicate from each sample:

$$\boldsymbol{Y}_1 := \begin{bmatrix} \boldsymbol{y}_{11}^T \\ \boldsymbol{y}_{21}^T \\ \vdots \\ \boldsymbol{y}_{n1}^T \end{bmatrix} .$$

Using $\tilde{\boldsymbol{Y}}_1$ to denote a column-scaled version of $\boldsymbol{Y}_1$, with each column of $\tilde{\boldsymbol{Y}}_1$ having sample mean of zero and sample variance of one. The $p \times p$ inter-analyte sample correlation matrix of $\boldsymbol{Y}_1$ is

$$\boldsymbol{R} := \frac{\tilde{\boldsymbol{Y}}_1^T \tilde{\boldsymbol{Y}}_1}{n-1}. \tag{10}$$

Bootstrapped data sets were generated to follow the estimated covariance structure of the true data, which can be usefully viewed as comprising two parts: the estimated inter-analyte covariance structure, as specified by $\boldsymbol{R}$ along with the $(\hat{v}_b^{(k)}, \hat{v}_e^{(k)})$; and the estimated within-analyte covariance structure, as specified by the relationship between $y_{ij}^{(k)}$, $b_i^{(k)}$ and $e_{ij}^{(k)}$ in (1).

Specifically, for the lth bootstrapped data set ($l = 1, \ldots, L$):

$$\begin{array}{rcl} \boldsymbol{V}_b & := & \text{diag}_k\left(\sqrt{\hat{v}_b^{(k)}}\right) \\ \boldsymbol{V}_e & := & \text{diag}_k\left(\sqrt{\hat{v}_e^{(k)}}\right) \\ \boldsymbol{b}_{[l]i} & \overset{\text{i.i.d. over } i}{\sim} & \text{Normal}\left(\boldsymbol{0}\ ,\ \boldsymbol{V}_b \boldsymbol{R} \boldsymbol{V}_b\right) \\ \boldsymbol{e}_{[l]ij} & \overset{\text{i.i.d. over } i,j}{\sim} & \text{Normal}\left(\boldsymbol{0}\ ,\ \boldsymbol{V}_e \boldsymbol{R} \boldsymbol{V}_e\right) \\ \boldsymbol{y}_{[l]ij} & := & \boldsymbol{b}_{[l]i} + \boldsymbol{e}_{[l]ij}\ , \end{array}$$

where $\text{diag}_k(\cdot)$ forms a $p \times p$ diagonal matrix from its argument with diagonal entries indexed by k. For the lth bootstrapped data set, the repeatability was estimated at each analyte using the ANOVA-based estimators, yielding estimates $\{\hat{R}_{[l]}^{(k)} : k = 1, \ldots, p\}$.

Bootstrap confidence intervals for $F(R)$ were calculated as follows. Denote the empirical distribution function of the lth bootstrap's repeatability estimates by $\hat{F}_{[l]}(R)$, i.e.

$$\hat{F}_{[l]}(R) \equiv \frac{1}{p} \sum_{k=1}^{p} I\left(R \leq \hat{R}_{[l]}^{(k)}\right)\ , \quad R \in [0, 1]. \tag{11}$$

Then the lower and upper bounds of the $1 - \alpha$ bootstrap confidence interval for $F(R)$ were the $\alpha/2$ and $1 - \alpha/2$ empirical quantiles of $\left\{\hat{F}_{[l]}(R) : l = 1, \ldots, L\right\}$.

Supporting Information Appendix C

This appendix contains the proof of **Proposition 2** which makes use of Lemma 2.

Consider the model for the mean measurement of treated and control samples,

$$y_i \;=\; \begin{cases} \mu_T + \varepsilon_i & \text{if sample } i \text{ from treated group } T \\ \mu_C + \varepsilon_i & \text{if sample } i \text{ from control group } C \end{cases} \tag{12}$$

where y_i is the measured concentration on the ith sample, the group means are denoted by μ_T and μ_C, and the residual terms ε_i are independent Normal random variables with mean zero and variance $V(\varepsilon_i) \equiv v_b + v_e$.

Lemma 2. *Sample size requirements for the t-test. Suppose under model (12) that it is required to have power at least $1-\beta$ to detect a standardized effect size of $\Delta \equiv \frac{|\mu_T - \mu_C|}{\sqrt{v_b}}$, at an analyte measured with repeatability $R \equiv \frac{v_b}{v_b+v_e}$, testing at significance level α. Then, a sample size of n ($n/2$ in each group) is large enough if and only if n satisfies*

$$1 - G_{n-2,\,\Delta\sqrt{nR/4}}\left[G^{-1}_{n-2,\,0}\left(1-\frac{\alpha}{2}\right)\right] \geq 1-\beta, \tag{13}$$

where $G_{\nu,\,\delta}$ denotes the distribution function of the non-central t-distribution with ν degrees of freedom (d.f.) and non-centrality parameter δ.

Proof. Under model (12) the t-statistic [33] (see his Section 4b.1) is

$$T \;:=\; \frac{(\bar{y}_T - \bar{y}_C)}{\sqrt{\frac{4(v_b+v_e)}{n}}} \div \sqrt{\frac{\frac{1}{n-2}\sum_{i=1}^{n}\left(y_i - \bar{y}_{g(i)}\right)^2}{v_b+v_e}} \tag{14}$$

where, e.g., $\bar{y}_T := \frac{2}{n}\sum_{\{i:g(i)=A\}} y_i$, so $E[\bar{y}_T] = \mu_T$ and $E[\bar{y}_C] = \mu_C$.

Under $H_0 : \mu_T = \mu_C$, T follows a t-distribution with $n-2$ d.f. (The first and second terms on the right-hand side of (14) are independently distributed Normal$(0,1)$ and χ^2_{n-2} respectively.)

Under the alternative hypothesis, $H_1 : \mu_T \neq \mu_C$, T follows a non-central t-distribution with

$n-2$ d.f. and non-centrality parameter

$$\delta := \frac{\mu_T - \mu_C}{\sqrt{\frac{4(v_b+v_e)}{n}}} \tag{15}$$

(The first and second terms on the right-hand side of (14) are independently distributed Normal$(\delta, 1)$ and χ^2_{n-2} respectively.)

The power is then*

$$\begin{aligned} \text{Power} &= Pr\left([\text{reject } H_0\,] \wedge\ [\text{sign}(T) = \text{sign}(\delta)\,]\ |\ H_1 \text{ true}\right) \\ &= Pr\left(T > G^{-1}_{n-2,\,0}\left(1-\frac{\alpha}{2}\right)\ \Big|\ Pr(T \le t) = G_{n-2,\,|\delta|}(t)\right) \\ &= 1 - G_{n-2,\,|\delta|}\left[G^{-1}_{n-2,\,0}\left(1-\frac{\alpha}{2}\right)\right]. \end{aligned}$$

Upon noting that $|\delta| \equiv \Delta\sqrt{nR/4}$, the result follows. □

Proposition 2. *Under model (12), suppose that a sample size n_0 ($n_0/2$ in each group) provides power $\geq 1-\beta$, testing at significance level α, to detect a standardized effect size of $\Delta \equiv \frac{|\mu_T - \mu_C|}{\sqrt{v_b}}$, at an analyte measured perfectly, i.e. with repeatability 1. Then $n := n_0/R$ provides power $\geq 1-\beta$, testing at significance level α, to detect Δ at an analyte measured with repeatability $R \equiv \frac{v_b}{v_b+v_e}$.*

Proof. Applying Lemma 2 under the assumed property of n_0 gives

$$1 - G_{n_0-2,\,\Delta\sqrt{n_0/4}}\left[G^{-1}_{n_0-2,\,0}\left(1-\frac{\alpha}{2}\right)\right] \geq 1-\beta. \tag{16}$$

Ghosh's [34] Theorem 8 states that

$$1 - G_{\nu,\,\delta}\left[G^{-1}_{\nu,\,0}\left(1-\frac{\alpha}{2}\right)\right] \text{ is increasing in } \nu \in (0,\infty) \tag{17}$$

*The usual definition of power is Pr(reject H_0 | H_1 true). In the current context—the reproducible detection of biological effects—we impose the further requirement that the true and estimated effects are of the same sign. This results in the definition of power as Pr([reject H_0] $\wedge$ [sign(Z) = sign(δ)] | H_1 true). The proposition is provable in the same way under either definition.

for fixed $\delta > 0$ and fixed $\alpha \in (0, 1)$.

Substituting $n = n_0/R$ gives:

$$
\begin{aligned}
1 - G_{n-2,\,\Delta\sqrt{nR/4}}\left[G^{-1}_{n-2,\,0}\left(1 - \frac{\alpha}{2}\right)\right] &= 1 - G_{n_0/R-2,\,\Delta\sqrt{n_0/4}}\left[G^{-1}_{n_0/R-2,\,0}\left(1 - \frac{\alpha}{2}\right)\right] \\
\text{[by (17), since } n_0/R \geq n_0\text{]} \quad &\geq 1 - G_{n_0-2,\,\Delta\sqrt{n_0/4}}\left[G^{-1}_{n_0-2,\,0}\left(1 - \frac{\alpha}{2}\right)\right] \\
\text{[by (16)]} \quad &\geq 1 - \beta
\end{aligned}
$$

and the result follows upon application of Lemma 2. □

Supplementary Figures

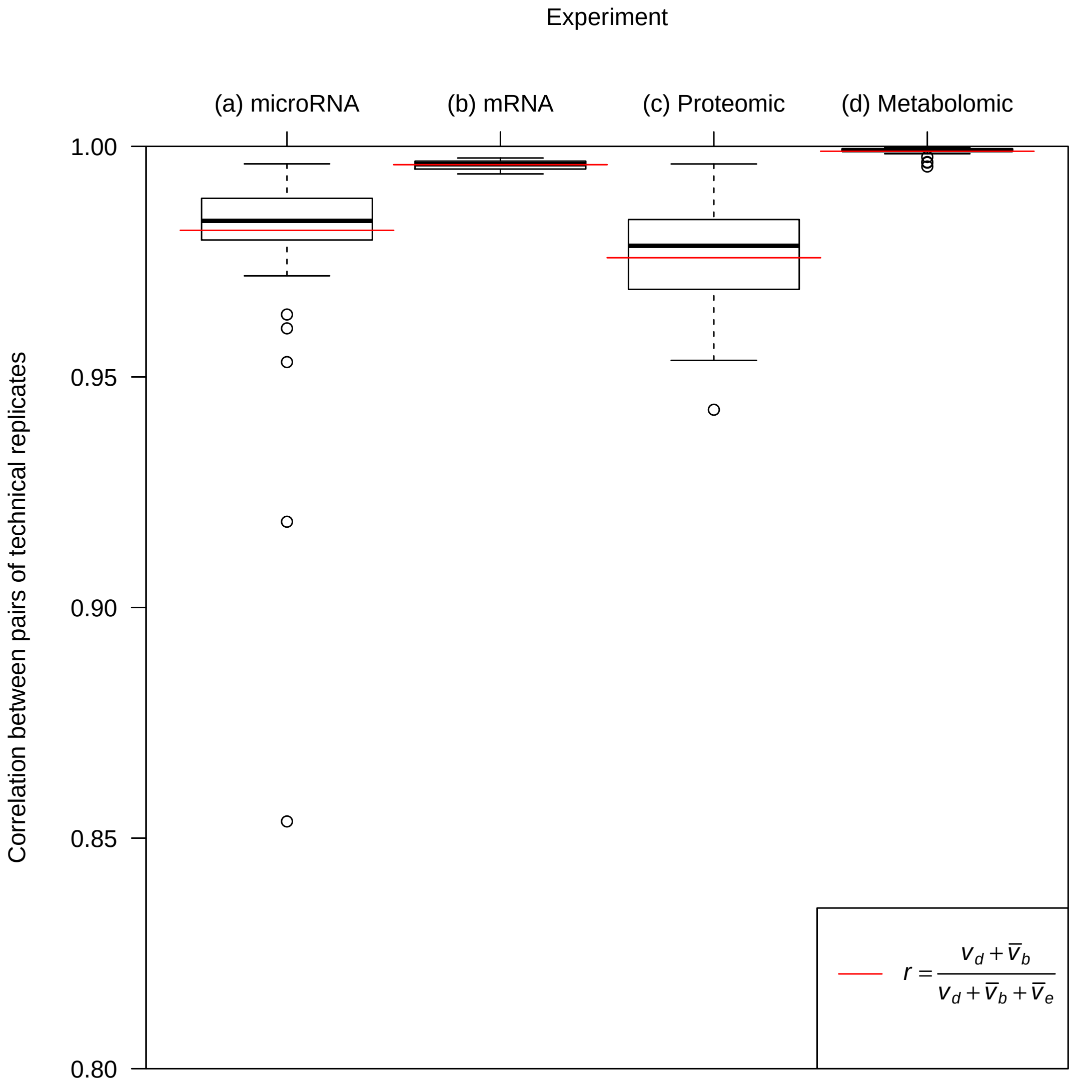

Supplementary Figure 1: Boxplots showing the distribution of values of r within each data set. Each value of r is calculated from a particular pair of technical replicates. The horizontal red line shows the plug-in estimate of $\frac{v_d+\bar{v}_b}{v_d+\bar{v}_b+\bar{v}_e}$ (the analyte-specific components of variance, $v_b^{(k)}$ and $v_e^{(k)}$, were estimated by ANOVA, and the resulting estimates averaged across analytes; analyte-specific means were estimated and then their sample variance was used to estimate v_d; the resulting estimates of v_d, $\bar{v}_b$ and $\bar{v}_e$ were then substituted into the ratio formula).

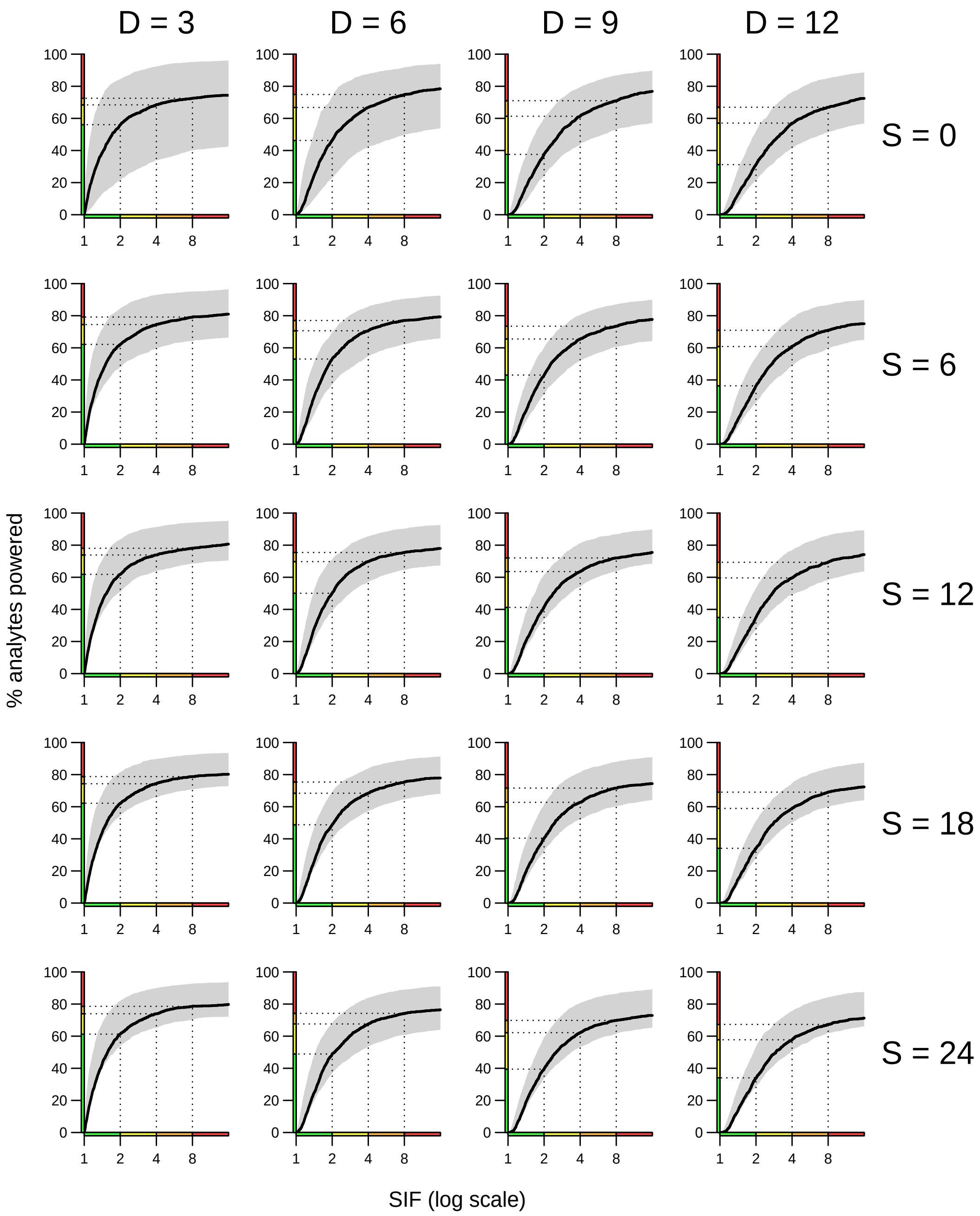

Supplementary Figure 2: Analysis of sub-sampled data sets for miRNA experiment (assay a). The plot for each sub data set is analogous to the bottom panels of **Figure 2**, as described in its legend and the main text. Each sub data set comprises both a number of samples, D, assayed in technical replicate (labelled top), and a number of samples, S assayed only once (labelled right).

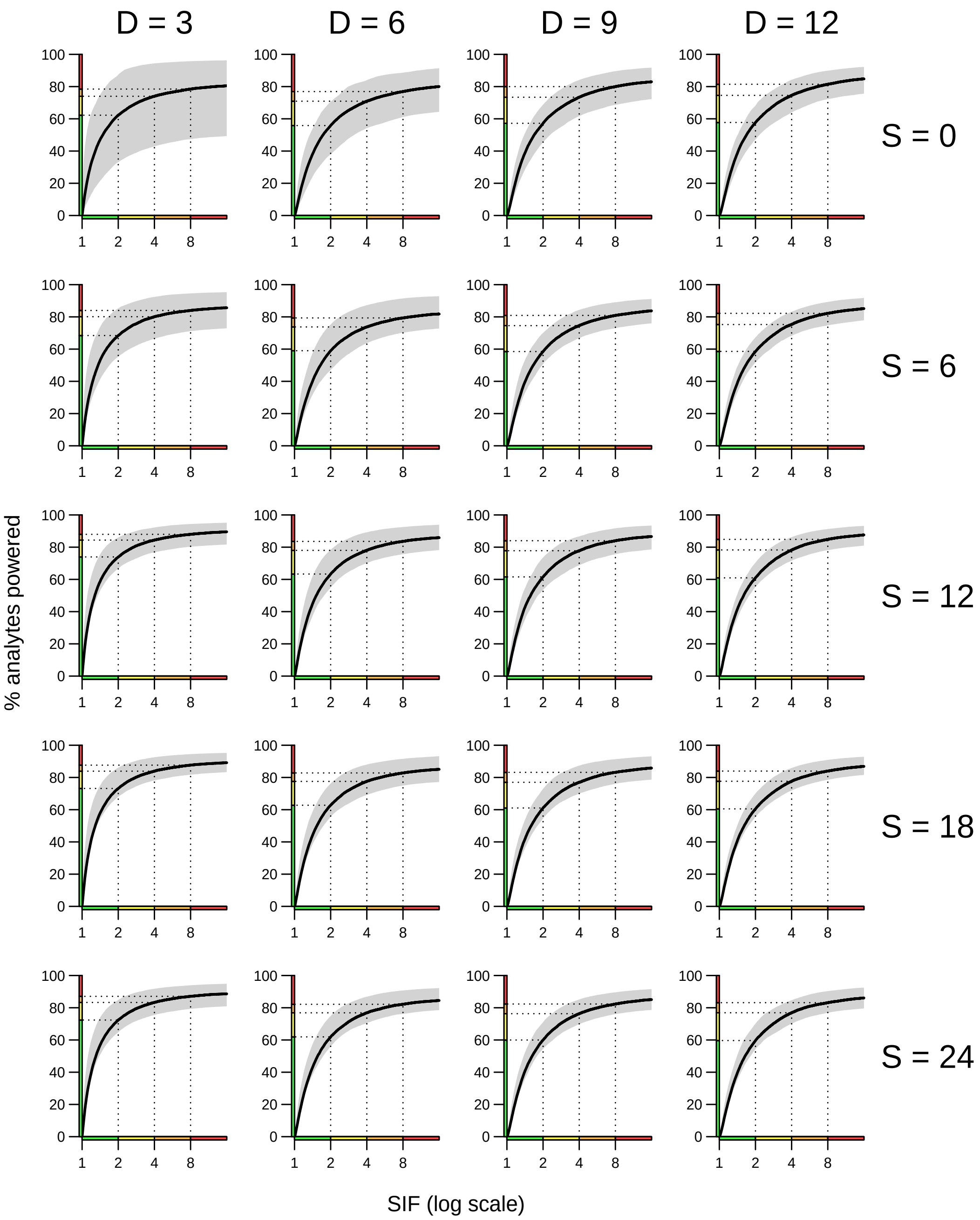

Supplementary Figure 3: Analysis of sub-sampled data sets for mRNA experiment (assay b). The plot for each sub data set is analogous to the bottom panels of **Figure 2**, as described in its legend and the main text. Each sub data set comprises both a number of samples, D, assayed in technical replicate (labelled top), and a number of samples, S assayed only once (labelled right).

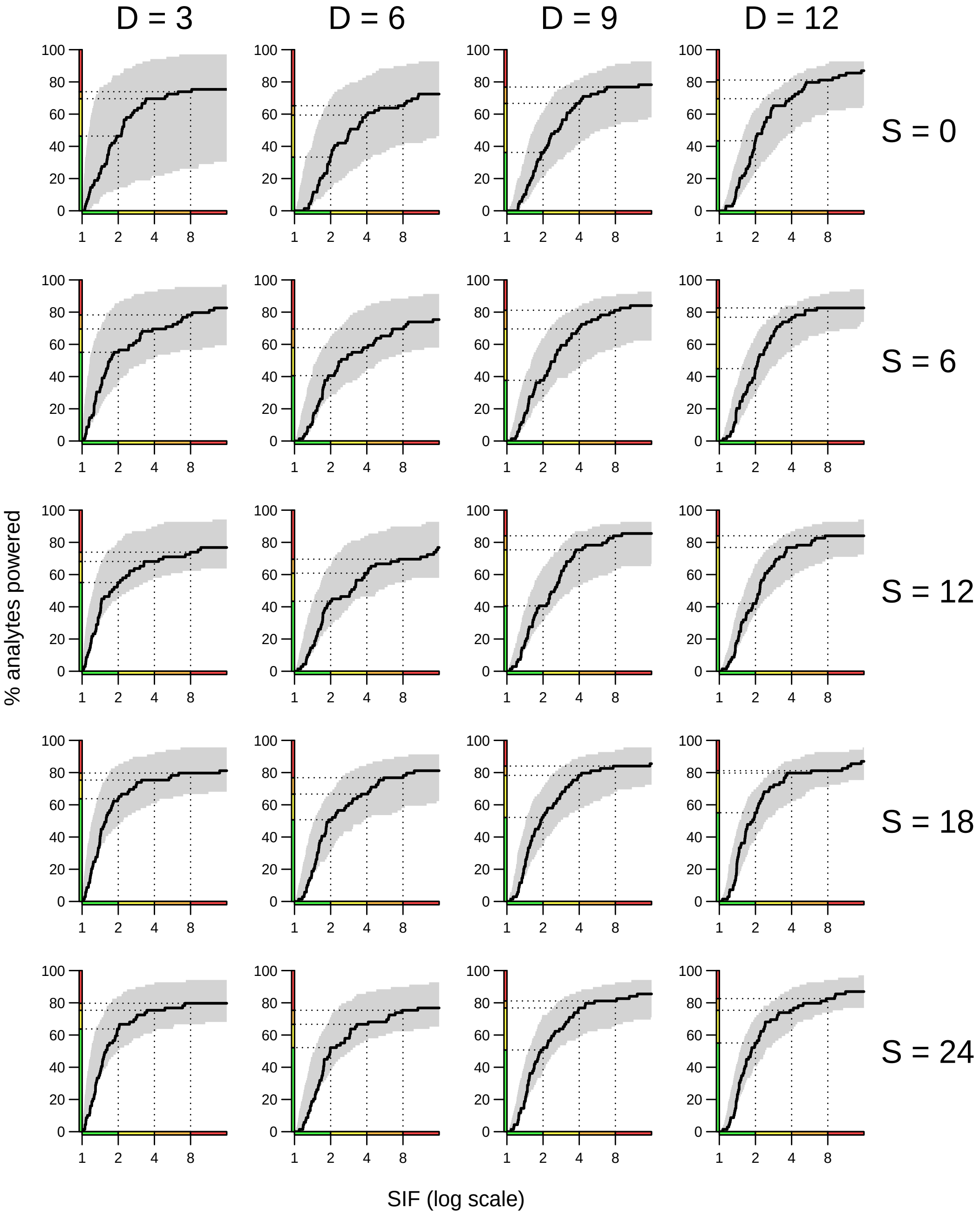

Supplementary Figure 4: Analysis of sub-sampled data sets for the proteomic experiment (assay c). The plot for each sub data set is analogous to the bottom panels of **Figure 2**, as described in its legend and the main text. Each sub data set comprises both a number of samples, D, assayed in technical replicate (labelled top), and a number of samples, S assayed only once (labelled right).

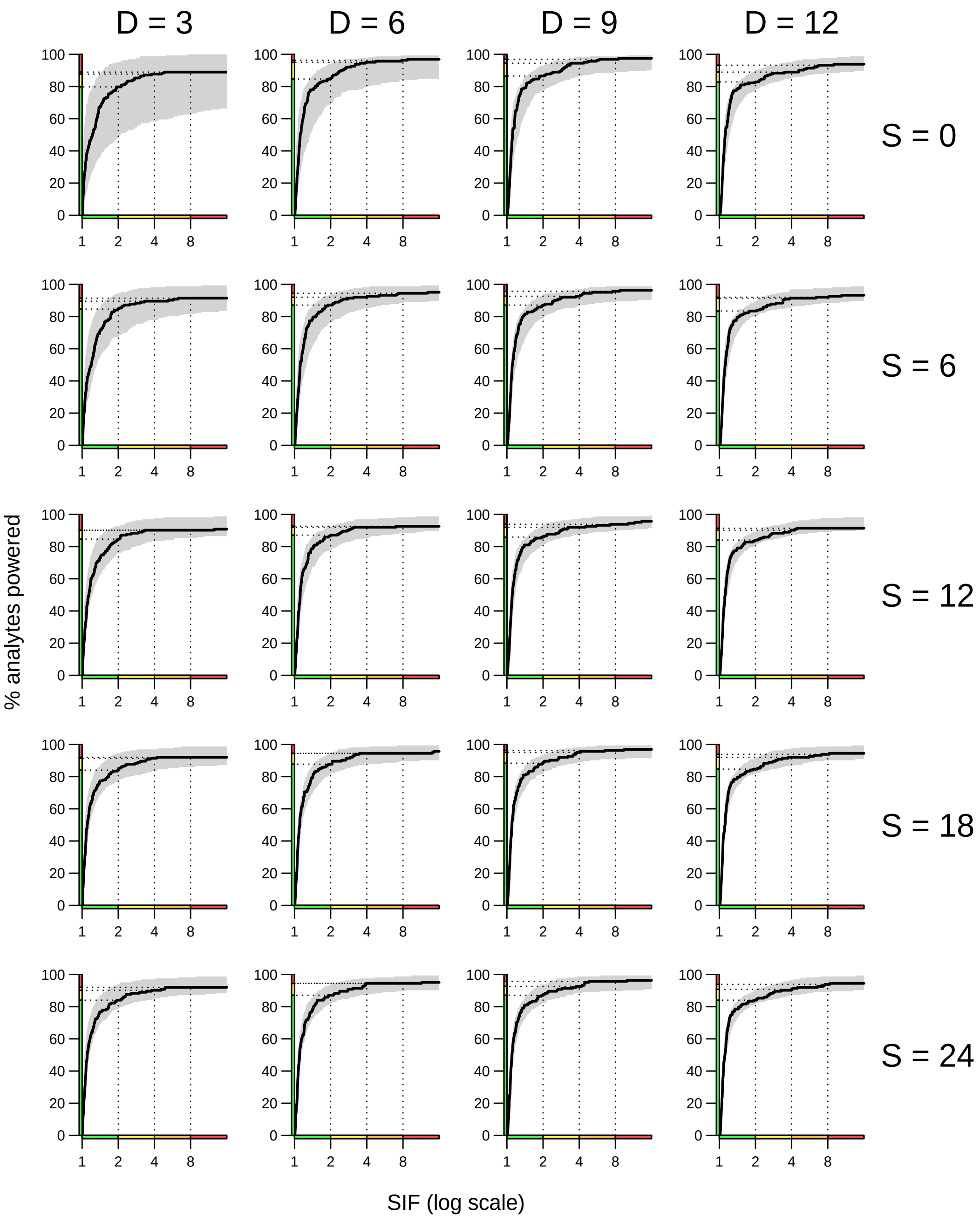

Supplementary Figure 5: Analysis of sub-sampled data sets for metabolomic experiment (assay d). The plot for each sub data set is analogous to the bottom panels of **Figure 2**, as described in its legend and the main text. Each sub data set comprises both a number of samples, D, assayed in technical replicate (labelled top), and a number of samples, S assayed only once (labelled right).
